# Conserved Disease Modules Extracted From Multilayer Heterogeneous Disease and Gene Networks for Understanding Disease Mechanisms and Predicting Disease Treatments

**DOI:** 10.3389/fgene.2018.00745

**Published:** 2019-01-18

**Authors:** Liang Yu, Shunyu Yao, Lin Gao, Yunhong Zha

**Affiliations:** ^1^School of Computer Science and Technology, Xidian University, Xi'an, China; ^2^Department of Neurology, Institute of Neural Regeneration and Repair, Three Gorges University College of Medicine, The First Hospital of Yichang, Yichang, China

**Keywords:** conserved disease modules, multilayer networks, gene networks, disease mechanisms, drug repositioning

## Abstract

Disease relationship studies for understanding the pathogenesis of complex diseases, diagnosis, prognosis, and drug development are important. Traditional approaches consider one type of disease data or aggregating multiple types of disease data into a single network, which results in important temporal- or context-related information loss and may distort the actual organization. Therefore, it is necessary to apply multilayer network model to consider multiple types of relationships between diseases and the important interplays between different relationships. Further, modules extracted from multilayer networks are smaller and have more overlap that better capture the actual organization. Here, we constructed a weighted four-layer disease-disease similarity network to characterize the associations at different levels between diseases. Then, a tensor-based computational framework was used to extract Conserved Disease Modules (CDMs) from the four-layer disease network. After filtering, nine significant CDMs were reserved. The statistical significance test proved the significance of the nine CDMs. Comparing with modules got from four single layer networks, CMDs are smaller, better represent the actual relationships, and contain potential disease-disease relationships. KEGG pathways enrichment analysis and literature mining further contributed to confirm that these CDMs are highly reliable. Furthermore, the CDMs can be applied to predict potential drugs for diseases. The molecular docking techniques were used to provide the direct evidence for drugs to treat related disease. Taking Rheumatoid Arthritis (RA) as a case, we found its three potential drugs Carvedilol, Metoprolol, and Ramipril. And many studies have pointed out that Carvedilol and Ramipril have an effect on RA. Overall, the CMDs extracted from multilayer networks provide us with an impressive understanding disease mechanisms from the perspective of multi-layer network and also provide an effective way to predict potential drugs for diseases based on its neighbors in a same CDM.

## Introduction

Complex diseases, such as cancers, diabetes mellitus, and cardiovascular disease, are caused by the combined effects of multiple genes, lifestyles and environmental factors (Craig, 2008), which makes it difficult to study and treat diseases. Studying the pathogenesis of diseases is critical to treat diseases because if it is controlled, the disease would be prevented (Last, 2000). Disease-disease relationship studies can help to understand the interrelationship between diseases and uncover the pathogenesis of diseases (Menche et al., 2015). Network theory is an available and useful solution for describing and analyzing the relationships between complex diseases (Barabási and Oltvai, 2004). To date, there are many network-based methods proposed to analyze diseases similarity. Menche et al. (2015) presented a new definition of module distance in incomplete interactome to predict disease-disease relationships. Zhou et al. (2014) constructed a human symptoms-based disease network using large-scale medical bibliographic records and the related Medical Subject Headings (MeSH) (Lowe and Barnett, 1994) metadata from PubMed (Wheeler et al., 2007). In 2007, Goh et al. (2007) gave the first disease network by connecting diseases that have common disease genes. Based on protein interactions and functional pathways, Liang et al. constructed a human disease network (HPDN) based on pathways to explore the potential relationships between diseases (Yu and Gao, 2017).

However, the biological data is incomplete (Menche et al., 2015), and the different levels of data used to construct disease relationships are usually interrelated (Gligorijević and Pržulj, 2015). That is to say, single-layer networks may not reveal the molecular mechanisms underlying the real systems because they simplify the varied nature of relationships (Kivelä et al., 2014). Moreover, only aggregating multiple types of interactions between diseases into a single network results in important temporal- or context-related information loss and may distort the actual organization (Rosvall et al., 2014; De Domenico et al., 2015). Therefore, in order to consider multiple types of interactions between diseases and the important interplays between layers, we use multilayer network model (Mucha et al., 2010; Cardillo et al., 2013; Nicosia et al., 2013; Radicchi and Arenas, 2013) to study the relevance between diseases from multiple perspectives. The detection of community structures is an essential method of network analysis and is key to understanding the structure of complex networks (Fortunato, 2010). Communities are topological groups of nodes which have more connections with each other than they are with the rest of nodes (Newman and Girvan, 2004; Porter et al., 2009; Fortunato, 2010). In recent years, researchers have proposed many methods to detect community structures on multilayer networks (Mucha et al., 2010; Li et al., 2011; Bazzi et al., 2014; Boccaletti et al., 2014; Liu et al., 2018). Li et al. presented a tensor-based computational framework for detecting recurrent dense subgraphs in multilayer weighted networks (Li et al., 2011). They applied their method to 130 co-expression networks and found 11,394 recurrent heavy subgraphs, i.e., densely connected node sets that consistently appear in the different layers. By validating against a large set of compiled biological knowledge bases, they showed their results are meaningful biological modules.

**Figure 1:**
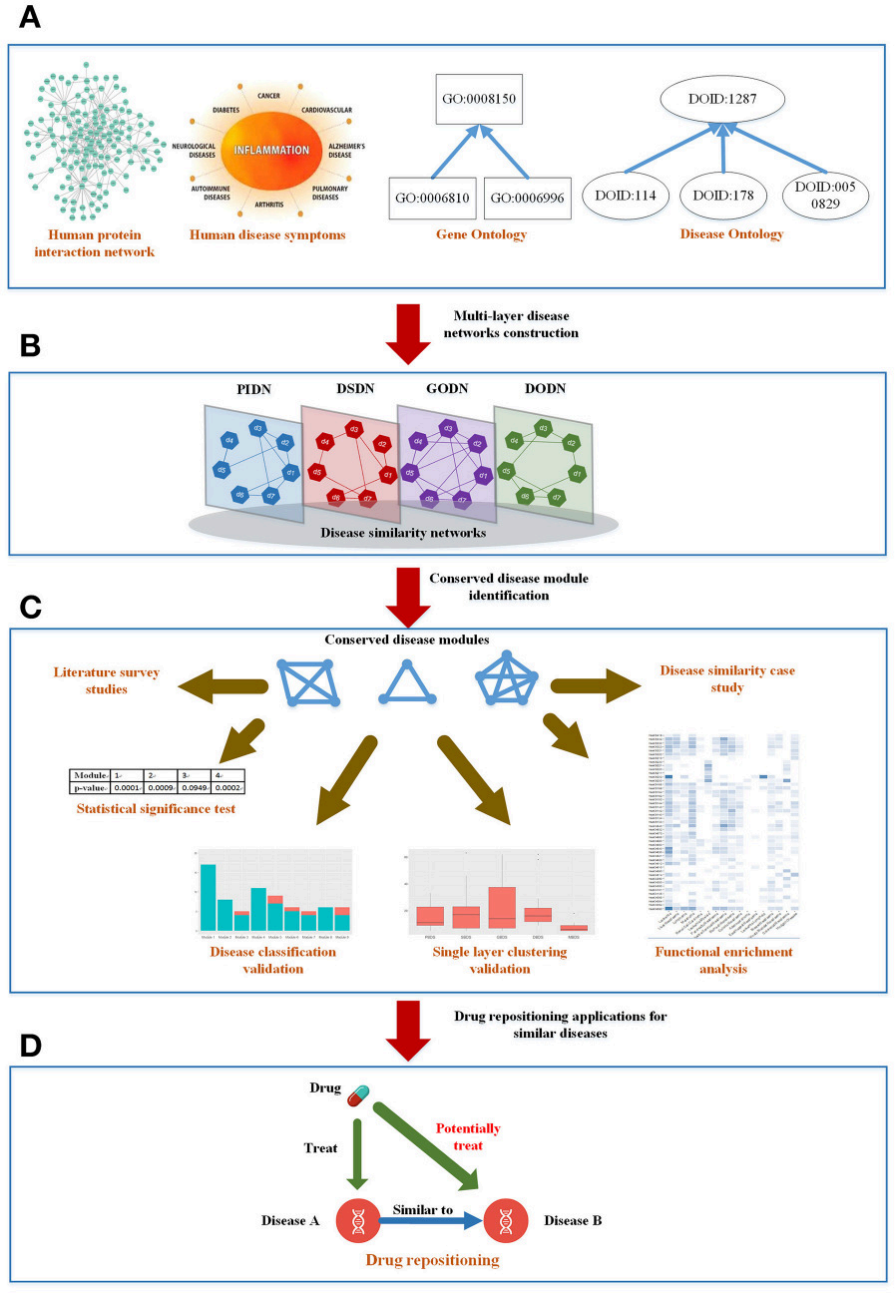
The mainframe of our work. **(A)** Four types of biological information related to diseases. **(B)** Construct a four-layer disease network based on the four types of data. **(C)** Extract conserved disease module (CDMs) from the four-layer network and verify them from different aspects. **(D)** Apply the conserved disease modules (CDMs) to drug repositioning.

Here, we constructed a weighted four-layer disease-disease similarity network to characterize the associations between diseases and detected community structures from the multilayer network to extract useful information, such as potential disease-disease associations. Further, based on the potential disease-disease associations, we tried to understand the underlying molecular mechanisms of diseases, and predicted new treatments for diseases. The tensor-based method (Li et al., 2011) was used here to identify significant and reliable disease-disease modules from our multilayer disease network. Because of the consistent appearances of the modules in all the layers, we named them as Conserved Disease Modules (CDMs). Figure 1 showed the whole framework of our method. We finally identified nine conserved disease modules (CDMs). After investigating these modules with the classification model in MeSH database, most of diseases in a same module belonged to a same classification. More importantly, as we expected, new disease-disease connections based on CDMs were found, which will help us to explore the unobserved molecular mechanisms of diseases and provided new treatments for them. We chose CDM 7 (classified as Cardiovascular Diseases) to predict potential drugs for Rheumatoid Arthritis (RA). With the help of molecular docking techniques, we predicted three potential drugs (Carvedilol, Metoprolol, and Ramipril) for RA. This results were also validated by literature.

## Results

### Constructing the Four-Layer Weighted Disease-Disease Similarity Network

#### Human Disease Network Based on Protein Interaction Network (PIDN)

The protein-protein interaction (PPI) network was got from ref (Menche et al., 2015), which consists of 13,460 genes and 141,296 interactions. In order to get the similarity between diseases based on the PPI network, we combined two datasets got from Online Mendelian Inheritance in Man (OMIM) database (Hamosh et al., 2005) and Genome-Wide Association Studies (GWAS) (Ramos et al., 2014) to get the disease-gene data, which includes 718 diseases and 22,410 genes (see Table S1). Then, we mapped the genes of each disease to the PPI network. Finally, based on the module distance definition (Menche et al., 2015) in incomplete networks, we calculated the similarity between disease pairs, and constructed the disease network PIDN. Here, nodes are diseases represented by their MeSH IDs (Mottaz et al., 2008). Weighted edges are correlations between disease genes based on module distance (Menche et al., 2015).

#### Human Disease Similarity Network Based on Symptoms (DSDN)

The symptom dataset of human diseases is based on the work of Zhou et al. (2014). Based on 322 symptom terms, they got a weighted disease-disease network. The nodes are diseases and the weighted edges are similarities between diseases. We further discarded the lower weighted edges to get a high confident network, which includes 1,596 nodes (diseases) and 133,106 edges (associations) (see Table S2).

#### Gene Ontology- and Disease Ontology-Based Disease Similarity Networks (GODN and DODN)

Gene Ontology (GO) (Ashburner et al., 2000) gives the definitions of concepts/classes for describing gene function, and associations between these concepts. It includes three categories: molecular function, cellular component, and biological process. Disease Ontology (DO) (Schriml et al., 2011) is a standardized ontology of human disease, which provides a comprehensive hierarchical controlled vocabulary for human disease including anatomy, cell of origin, infectious agent, and phenotype axioms. We evaluated the relationships between diseases based on the terms in GO and DO separately to get two disease similarity networks GODN (see Table S3) and DODN (see Table S4). The details of constructing networks are shown in Method section.

#### Four-Layer Weighted Disease-Disease Similarity Network

We selected the common nodes (diseases) from PIDN, DSDN, GODN, and DODN. They have 399 overlapped diseases. Then, based on the 399 diseases, we extracted four spanning subgraphs, which consist of the final four-layer disease-disease network.

### Extracting Conserved Disease Modules (CDMs) From the Four-Layer Weighted Disease Similarity Network

In real-world networks, weights on edges characterize the strength, intensity or capacity between nodes (Wasserman and Faust, 1994; Barrat et al., 2004). It is obvious that weighted networks describe information more accurate than their unweighted counterparts. Further, studies showed that in real-world networks, nodes tended to cluster into densely connected subnetworks (Watts and Strogatz, 1998; Louch, 2000; Snijders, 2001). In order to analyze the four-layer weighted disease network further, we used the tensor-based computational framework proposed by Li et al. (2011) to extract conserved disease modules (CDMs) from the multi-layer network. Li's method (Li et al., 2011) mined recurrent heavy subgraphs (RHSs) from multiple weighted networks. Here, we named RHSs as conserved disease modules (CDMs). The definition of CDM is based on that of heavy subgraphs (HS), a subset of heavily interconnected nodes in a single network. The nodes of a CDM are the same in each layer, but the edge weights may vary in different layers. The calculation details are shown in Method section. Finally, we got nine CDMs shown in Table 1.

**Table 1:** The classifications of the nine conserved disease modules in MeSH.

**ID of CDM**	**Diseases in CDM**	**Classes in MeSH**	**Number of diseases**
CDM 1	Leukemia, Liver Neoplasms, Lymphoma, Leukemia (Myeloid, Acute), Melanoma, Carcinoma(Renal Cell), Pancreatic Neoplasms, Uterine Cervical Neoplasms, Stomach Neoplasms, Colonic Neoplasms, Adenocarcinoma, Esophageal Neoplasms, Leukemia(Lymphoid), Breast Neoplasms, Urinary Bladder Neoplasms, Colorectal Neoplasms, Hodgkin Disease	Neoplasms	17
CDM 2	Diabetes Mellitus, Hyperglycemia, Hyperinsulinism, Obesity, Glucose Intolerance, Metabolic Diseases, Metabolic Syndrome X, Hyperlipidemias	Nutritional and Metabolic Diseases	8
CDM 3	Glomerulonephritis, Proteinuria, ***Lupus Erythematosus*****(*****Systemic*****)**, Nephrosis, Glomerulonephritis(IGA)	Male Urogenital Diseases	5
CDM 4	Neuromuscular Diseases, Amyotrophic Lateral Sclerosis, Motor Neuron Disease, Peripheral Nervous System Diseases, Hereditary Sensory and Motor Neuropathy, Movement Disorders, Epilepsy, Brain Diseases, Charcot-Marie-Tooth Disease, Central Nervous System Diseases, Huntington Disease	Nervous System Diseases	11
CDM 5	Thrombocytopenia, Blood Platelet Disorders, Hemorrhagic Disorders, Hemolytic-Uremic Syndrome, Hematologic Diseases, Anemia(Hemolytic), Anemia(Aplastic), Agammaglobulinemia, ***Colitis***(***Ulcerative***)	Hemic and Lymphatic Diseases	9
CDM 6	Pulmonary Fibrosis, Bronchiolitis Obliterans, Pulmonary Disease(Chronic Obstructive), Pulmonary Alveolar Proteinosis, ***Celiac Disease***, Bronchiectasis	Respiratory Tract Diseases	6
CDM 7	Cardiomyopathy(Dilated), Cardiomyopathy(Hypertrophic), Cardiomyopathies, Heart Failure, ***Rheumatoid arthritis***	Cardiovascular Diseases	5
CDM 8	Metabolism, Carbohydrate Metabolism, Metal Metabolism, Down Syndrome, Mental Retardation(X-Linked), Glycogen Storage Disease	Congenital, Hereditary, and Neonatal Diseases and Abnormalities	6
CDM 9	***Sarcoidosis***, Uveitis, Glaucoma, ***Cranial Nerve Diseases***, Retinitis Pigmentosa, Retinal Diseases	Eye Diseases	6

*Diseases with different classification are marked as bold italic in the second column*.

### Classification of the Nine Conserved Disease Modules

For the nine CDMs, their average size is 8.2 diseases. According to disease classification model in Medical Subject Headings (MeSH) (Mottaz et al., 2008), we made a classification for the nine CDMs. For a CDM, if more than 60% of its diseases belong to a same class *F* in MeSH, this CDM is marked as class *F*. The classification results are shown in the third column of Table 1 and the diseases with different classifications are marked as bold italic in the second column of Table 1. For example, CDM 3 includes five diseases and the classification of Lupus Erythematosus (Systemic) is different from other four diseases. Therefore, it is marked as bold italic in Table 1.

**Figure 2:**
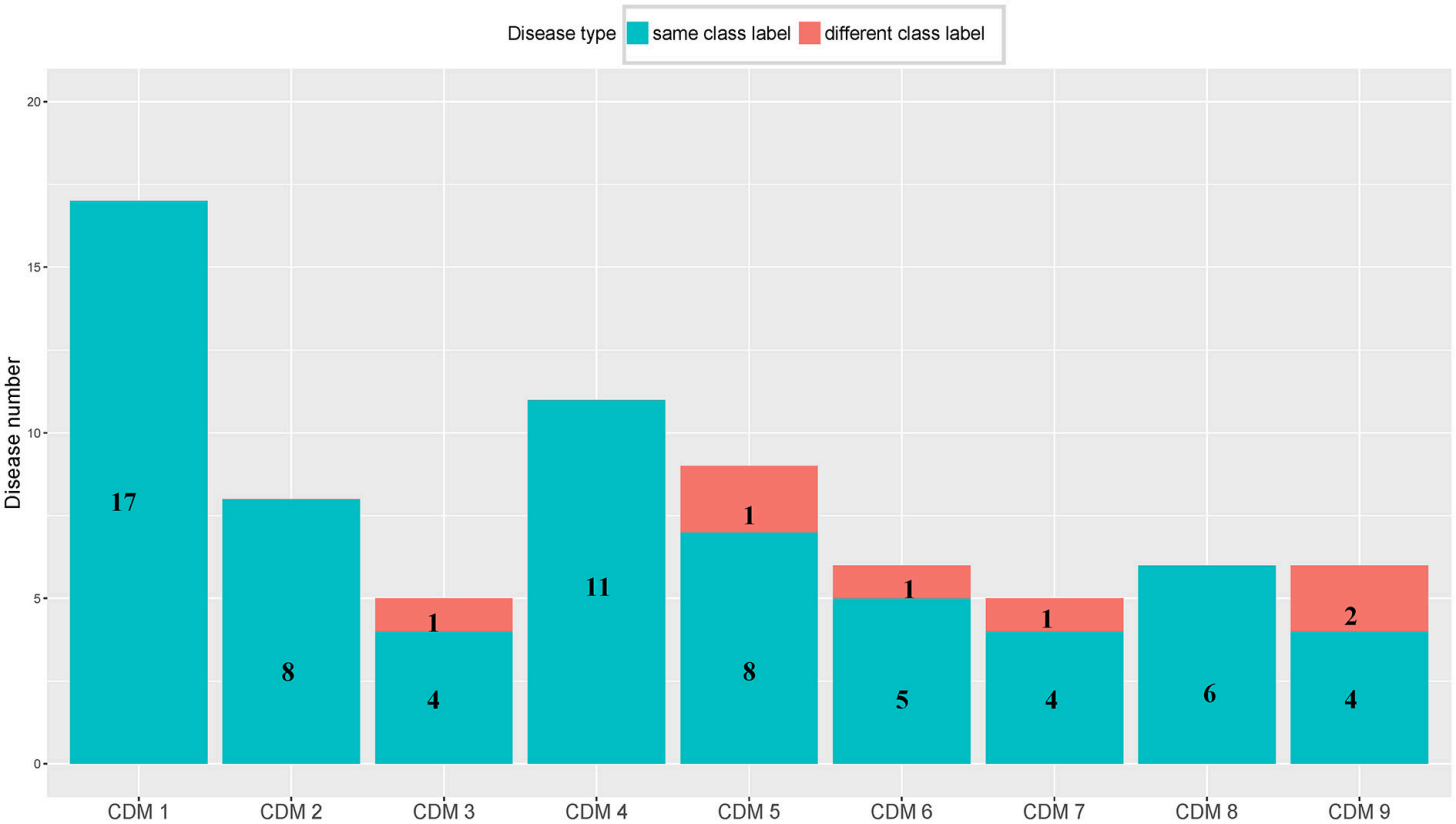
The comparison between the number of diseases with the same class label and the number of diseases with different class labels. The blue bar and the number on it represent the number of diseases having the same class with its CDM. The number of remaining diseases are marked on the red bar.

From Table 1, we can get five CDMs including diseases with different class labels. Figure 2 gives the further analyzed results. For each CDM, the figure gives the comparison between the number of diseases with the same classification and the number of diseases with different classifications. From Figure 2, we can find that our method not only can find the strong connections between diseases with the same classification, but also can predict the potential relationship between diseases.

### Statistical Significance of the Nine Conserved Disease Modules

To assess the statistical significance of the nine conserved disease modules, we respectively, generated four types of random networks based on PIDN, DSDN, GODN, and DODN. For each type of network, 1,000 random networks were generated, which maintained the degree distribution of the original network. Using the same method (Li et al., 2011), we did not find any conserved disease module. In addition, we also made an analysis based on the disease similarity network got from van Driel et al. (2006), which used text mining to classify human diseases contained in OMIM (Hamosh et al., 2005). Based on each of the nine CDMs, we randomly selected a module with the same size from the network. And then we summed the edge weights in the random module to make a comparison with that of the real CDM. We repeated this process 10,000 times to get the *p*-value for each of the nine CDMs. The results are shown in Table 2. From Table 2, we can find that the *p*-values of all the nine CDMs are significant, i.e., *p* < 0.1 and four of them are lower than 0.001.

**Table 2:** The *p*-values of the nine CDMs compared with random modules.

**ID of CDM**	**1**	**2**	**3**	**4**	**5**	**6**	**7**	**8**	**9**
*p*-value	0.0001	0.0009	0.0949	0.0002	0.0753	0.0422	0.0250	0.0009	0.0158

### Comparison With Single Layer Networks

**Figure 3:**
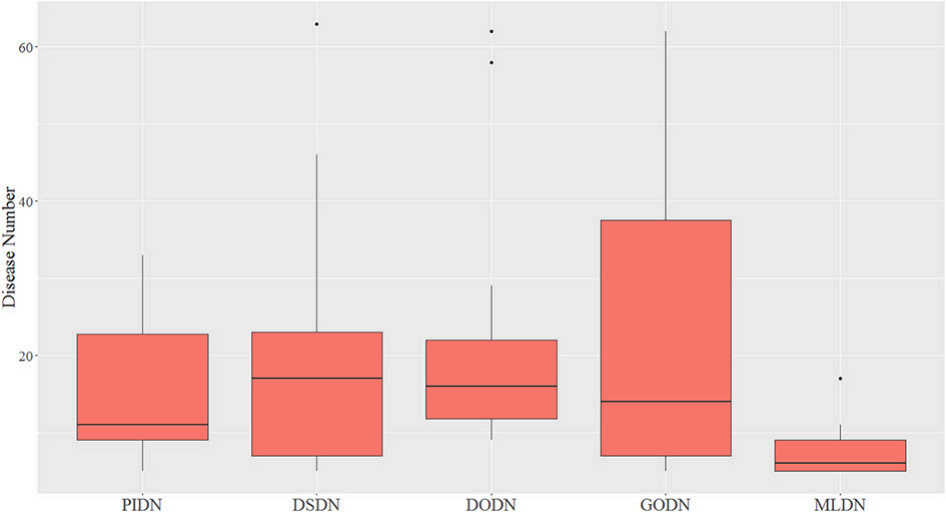
The size distribution of modules identified from each single layer network and our multi-layer network.

We also made a comparison between our multi-layer network and single layer networks. Here, ClusterONE algorithm (Nepusz et al., 2012) was used to do clustering analysis for the four single layer disease networks: PIDN, DSDN, DODN, and GODN. The size distribution of modules identified from each single layer network are shown in Figure 3. We also gave the size distribution of CMDs got from our multi-layer network marked as MLDN (Multi-layer Disease Network).

From Figure 3, we can see the sizes of disease modules got from single layer networks are almost all larger than that of modules got from our multi-layer network. This result is consistent with the findings of Domenico's group (De Domenico et al., 2015). Using a multi-layer network to characterize the relationship between diseases, we can get smaller disease modules with more overlap that better capture the actual disease-disease relationships. The major reasons are maybe that the biological data is incomplete, such as the interactome and the disease gene list (Hart et al., 2006; Wass et al., 2011), and single layer networks only consider single-dimensional biological information, which may introduce false positive data. Multi-layer networks integrate multi-dimensional related information, which are complementary and can eliminate the uncertainty caused by single-dimensional data. Therefore, the modules extracted from multi-layer networks are smaller and more accurate. Additionally, based on the multilayer network, some potential disease conserved modules can be identified, such as CDM 3, CDM 5, CDM 6, CDM 7, and CDM 9 (shown in Table 1). They all contain at least one disease with a different classification. Taking CDM 6 as an example, it includes six diseases: Pulmonary Fibrosis, Bronchiolitis Obliterans, Pulmonary Disease (Chronic Obstructive), Pulmonary Alveolar Proteinosis, ***Celiac Disease***, Bronchiectasis. For Celiac Disease, it is a serious genetic autoimmune disease. The other five diseases belong to Respiratory Tract Diseases in MeSH database. If we only extracted modules from PIDN, DSDN, or the common subgraph of four networks, CMD 6 will not be found. The main reason is that we have constructed a weighted four-layer disease network instead of just getting the common subgraph of four single-layer networks, and we chose the tensor-based method (Li et al., 2011) to identify the disease conserved modules. This method is suitable for clustering analysis of weighted multi-layer networks (Li et al., 2011).

### KEGG Pathway Functional Enrichment Analysis and Investigation of Pathogenesis

In this section, we further performed Kyoto Encyclopedia of Genes and Genomes (KEGG) (Kanehisa and Goto, 2000) pathway enrichment analysis on diseases and their related genes. KEGG (http://www.genome.jp/kegg/) is an encyclopedia of genes and genomes (Kanehisa and Goto, 2000). Its primary objective is assigning functional meanings to genes and genomes both at the molecular and higher levels. We applied DAVID (Dennis et al., 2003), which is a functional annotation tool, to make KEGG pathway enrichment analysis. Based on disease gene list got from OMIM, we can obtain disease's enriched KEGG pathways (*p* ≤ 0.01) for each disease in a given CDM by using DAVID.

**Figure 4:**
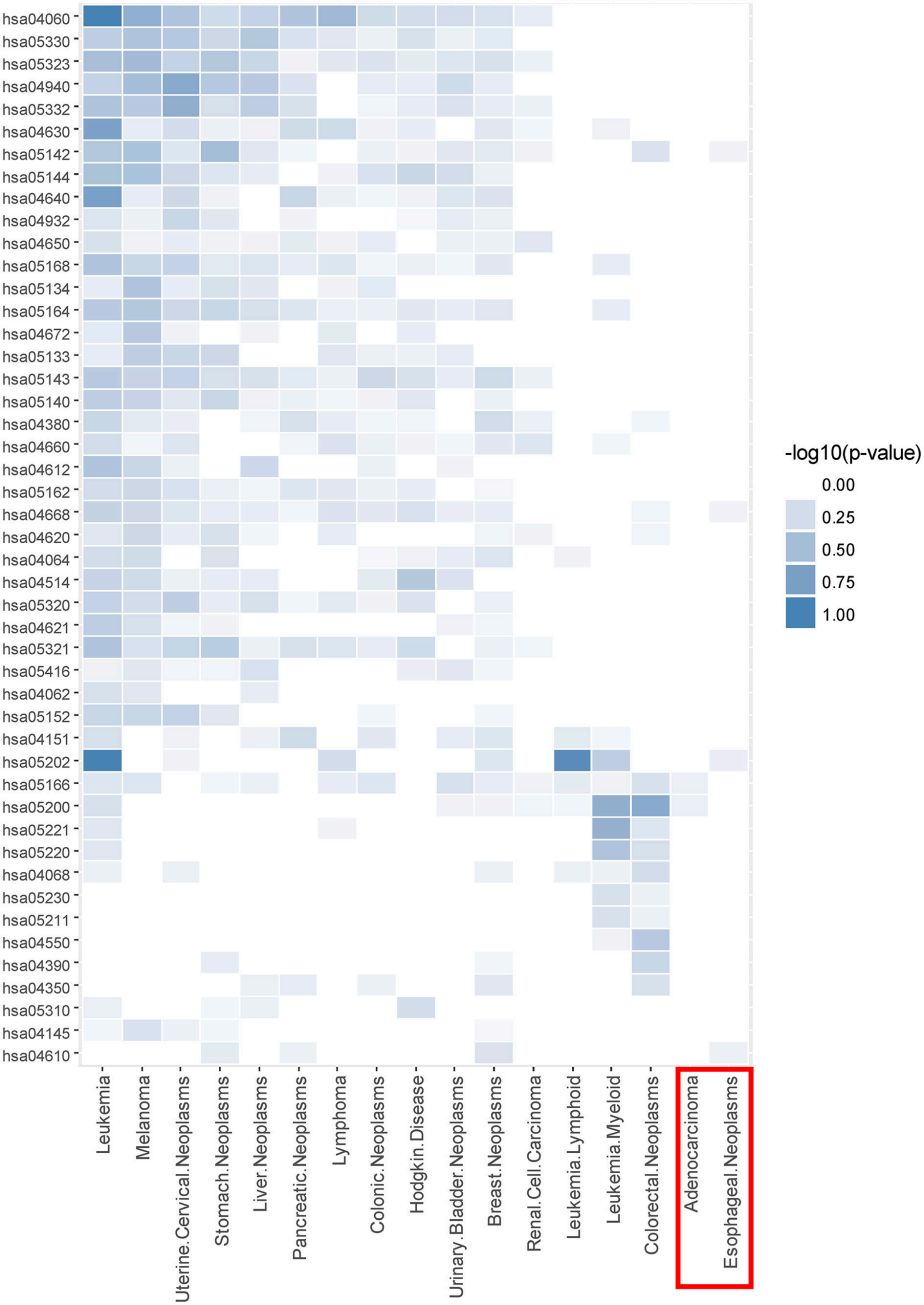
The pathway enrichment analysis of diseases in conserved disease module 1 (CDM 1). The horizontal axis indicates 17 diseases and the vertical axis represents their enriched 47 pathways. The colors of small bricks from white to steel blue represent the *p*-values with negative log conversion with the maximum and minimum normalization. The greater the value, the more significant the enrichment.

Taking CDM 1 as an example, Figure 4 gives the relationship analysis between 17 diseases in CDM 1 based on their corresponding 47 pathways. From Figure 4, we can see these 17 diseases have a great pathway overlapping. These pathways include some important ones that associated with cancer, such as “hsa05202: Transcriptional misregulation in cancer,” “hsa05200: Pathways in cancer,” “hsa04060: Cytokine-cytokine receptor interaction,” and “hsa04630: Jak-STAT signaling pathway,” which is consistent with that all the diseases in CDM 1 belong to “Neoplasms” in MeSH (see Table 1).

In Figure 4, Adenocarcinoma and Esophageal Neoplasms (marked by red solid rectangle) seem to enrich with few pathways. The reason is maybe that we cannot get more genes related to them at present. In fact, based on multidimensional information we used in this paper, we can find their strong relationship with other diseases, and group them together, which indicates that our multi-layer network method can help to complement the incompleteness of one-dimensional biological data.

### Analyzing Disease Genes With the Maximum Frequency

We tried to analyze the pathogenesis of diseases through their similar neighbor diseases. Each disease has a related gene list. For a conserved disease module, we count the frequency of each gene appearing in all its gene lists. For example, CDM 1 contains 17 diseases, so it has 17 gene lists. If one gene appears in all the 17 gene lists, its frequency is 17. For each conserved disease module, we chose its genes with the max frequency. The results are shown in Table 3. Those genes with the maximum frequency in a module maybe be the potential targets of diseases or related with the targets of diseases in the module.

**Table 3:** The gene lists with the maximum frequency in each conserved disease modules.

**ID of CDM**	**Number of diseases**	**Max frequency of genes**	**Genes with the maximum frequency**
CDM 1	17	10	TNF
CDM 2	8	6	REN
CDM 3	5	2	CTLA4, FCGR3B, IL6, APOE, MMP9, PTX3, F3, AGT, HPX, CTGF, CCL2, ACE, ADM
CDM 4	11	6	NEFL
CDM 5	9	5	ADAMTS13, ITGA2B, ITGB3
CDM 6	6	3	IL6, IFNG, IL10
CDM 7	5	4	PLN, MYH6, MYH7
CDM 8	6	3	LAMP2, HYAL1, GBE1, SGSH, ATP7A, PRKAG2, AGL, HGSNAT, PDHA1, PDHB, IDS, IDUA
CDM 9	6	3	RLBP1, MYOC, LOXL1

We still took CDM 1 as our case for further analysis. In CDM 1, it contains 17 diseases, and TNF (tumor necrosis factor) is found having the maximum frequency 10 in all the 17 diseases. That is to say, TNF is the causal gene of 10 diseases in CDM 1. For the other 7 diseases (Lymphoma, Colorectal Neoplasms, Esophageal Neoplasms, Hodgkin Disease, Leukemia Lymphoid, Leukemia Myeloid, and Adenocarcinoma) in CDM 1, TNF maybe their potential causal gene or have close connections with their casual genes in protein-protein interaction (PPI) network, which will be helpful for studying the pathogenesis of these diseases. Tumor necrosis factor (TNF or TNF-α) is a cell signaling protein (cytokine) involved in early inflammatory events. It effects on lipid metabolism, coagulation, insulin resistance, and the function of endothelial cells lining blood vessels (Vassalli, 1992). Drugs that block the action of TNF have been shown to be beneficial in reducing the inflammation in inflammatory diseases, such as Crohn's disease and Rheumatoid Arthritis (Raza, 2000).

In fact, four of the seven diseases, Lymphoma, Colorectal Neoplasms, Esophageal Neoplasms, and Hodgkin Disease, significantly enrich with “hsa04668: TNF signaling pathway” according to the above analysis in Figure 4. TNF can induce a wide range of intracellular signal pathways including apoptosis and cell survival as well as inflammation and immunity. For the remaining three diseases, Leukemia (Lymphoid), Leukemia (Myeloid, Acute) and Adenocarcinoma, we find at least one of their casual genes have strong connections with TNF in PPI network (Greene et al., 2015).

### Verify Disease Relationships in a Same CDM With Different Classifications

Our method found five significant conserved disease modules including diseases with different classifications in MeSH database (shown in Table 1). In this section, we took CMD 3 as an example, which is composed of five diseases: Glomerulonephritis, Proteinuria, ***Lupus*** ***Erythematosus***, Nephropathy, and Glomerulonephritis(IGA) (A chronic form of glomerulonephritis). In the five diseases, except for Lupus Erythematosus, the other four diseases are all male urogenital diseases. We tried to find the potential connections between Lupus Erythematosus, and the other four diseases.

All the disease-related treatment drugs were downloaded from Comparative Toxicogenomics Database (CTD) (Davis et al., 2012) and those drugs marked as “T” (therapeutic) are chosen, which means these drugs are used to treat its corresponding diseases (Davis et al., 2012). For any disease pair *d*_1_ and *d*_2_ in CDM 3, their related drug sets are denoted as *Drug*_*Therapeutic*_*d*_1__ and *Drug*_*Therapeutic*_*d*_2__, respectively. We used Jaccard index (Jaccard, 1912) to calculate the similarity between *d*_1_ and *d*_2_ shown as following:

(1) $$J(d_{1},d_{2})=\frac{Drug\_Therapeutic_{d_{1}}\cap Drug\_Therapeutic_{d_{2}}}{Drug\_Therapeutic_{d_{1}}\cup Drug\_Therapeutic_{d_{2}}}$$

We found Lupus Erythematosus has high similarity with other diseases in CDM 3. The Jaccard indexes between Lupus Erythematosus and other two diseases, Glomerulonephritis, and Nephrosis, are both 0.4. The results indicate that Lupus Erythematosus shares a lot of drugs with other diseases in CDM 3 for treatment.

In fact, many reports pointed out that Lupus Erythematosus has a strong correlation with other diseases in CDM 3. For example, in 2004, Weening et al. (2004) pointed out Glomerulonephritis and Lupus Erythematosus should be classified in a same class. Machado et al. (2005) reported a case of a 10-years-old girl with Systemic Lupus Erythematosus (SLE) presenting with Nephrotic Syndrome and Membranous Glomerulopathy.

### Application of Conserved Disease Modules in Drug Repositioning

#### Scoring Drugs Based on Diseases in Conserved Disease Modules

Drug repositioning is a strategy to identify new therapeutic applications for existing drugs (Ashburn and Thor, 2004). For a conserved disease module, drugs that were used to treat some of these diseases were then regarded as potential drugs for the other diseases in the same disease module (Dudley et al., 2011). Based on the assumption, we tried to predict reusable drugs for the diseases in a same conserved disease module. Firstly, we chose the related drugs for each conserved disease module through combining all the drugs related to the diseases in it. Drugs marked as “T” (therapeutic) were chosen from the CTD database and each of them was scored by the following formula:

(2) $$Drug\_score=\frac{n_{T}}{N}$$

Where *N* indicated the total number of diseases in a conserved disease module; *n*_*T*_ indicated the number of diseases related with this drug in this conserved disease module.

Here, we took CDM 7 as an example. Table 4 shows the scoring drugs of CDM 7. CDM 7 contains five diseases: Cardiomyopathies (CM), Dilated Cardiomyopathy (DCM), Hypertrophic Cardiomyopathy (HCM), Heart Failure (HF), and Rheumatoid Arthritis (RA). The drugs with *Drug*_*score* ≥ 0.6 were selected. In other words, we believed that drugs that are associated with more than 60% of the diseases are also likely to be effective for treating other diseases in the same module. For each drug, if it has a “T” (therapeutic) connection (Davis et al., 2012) with a disease in CTD database, it will be marked as “1” in the corresponding position in Table 4, otherwise it will be marked as “0.”

**Table 4:** Drugs with *Drug*_*score*≥0.6 for CDM 7 based on disease-drugs pairs in CTD.

**No**	**Drug name**	**MeSH ID**	**CM**	**DCM**	**HCM**	**HF**	**RA**	***Drug_score***
1	Losartan	D019808	1	1	1	1	1	1
2	Resveratrol	C059514	1	1	1	1	1	1
3	Carvedilol	C043211	1	1	1	1	0	0.8
4	Angiotensin-converting enzyme inhibitors	D000806	1	0	1	1	1	0.8
5	Metoprolol	D008790	1	1	1	1	0	0.8
6	Ramipril	D017257	1	1	1	1	0	0.8
7	Azathioprine	D001379	1	0	1	1	1	0.8
8	Prednisone	D011241	1	0	1	1	1	0.8
9	Benazepril	C044946	1	0	1	1	0	0.6
10	Enalapril	D004656	1	0	1	1	0	0.6
11	Dobutamine	D004280	1	1	1	0	0	0.6
12	Spironolactone	D013148	1	0	1	1	0	0.6
13	Amiodarone	D000638	1	1	1	0	0	0.6
14	Nifedipine	D009543	1	1	1	0	0	0.6
15	Torsemide	C026116	1	0	1	1	0	0.6
16	Candesartan cilexetil	C077793	1	0	1	1	0	0.6
17	Morphine	D009020	1	0	1	0	1	0.6
18	Dipyridamole	D004176	1	0	1	1	0	0.6
19	Hydralazine	D006830	1	0	1	1	0	0.6
20	Ceftriaxone	D002443	1	0	1	0	1	0.6
21	Dihydralazine	D004078	1	0	1	1	0	0.6
22	Diuretics	D004232	1	0	1	1	0	0.6
23	Enoximone	D017335	1	0	1	1	0	0.6
24	Rosiglitazone	C089730	0	1	1	0	1	0.6
25	Protein kinase inhibitors	D047428	0	1	1	1	0	0.6
26	Quinapril	C041125	0	1	1	1	0	0.6
27	Candesartan	C081643	0	1	1	1	0	0.6
28	Sulfinpyrazone	D013442	0	1	1	0	1	0.6
29	Drugs, Chinese herbal	D004365	0	0	1	1	1	0.6
30	Plant extracts	D010936	0	0	1	1	1	0.6

*The forth to eighth columns represent the therapeutic relationships between drugs and diseases. If a drug and a disease have a therapeutic relationship in CTD database, the value of the corresponding intersection is “1.” otherwise the value is “0.” The last column indicates that the Drug_score for each drug in the CDM 7 based on the formula (2). CM, cardiomyopathies; DCM, dilated cardiomyopathy, HCM, hypertrophic cardiomyopathy; HF, heart failure; RA, rheumatoid arthritis*.

#### Verifying Potential Drugs Based on Molecular Docking Experiments

**Figure 5:**
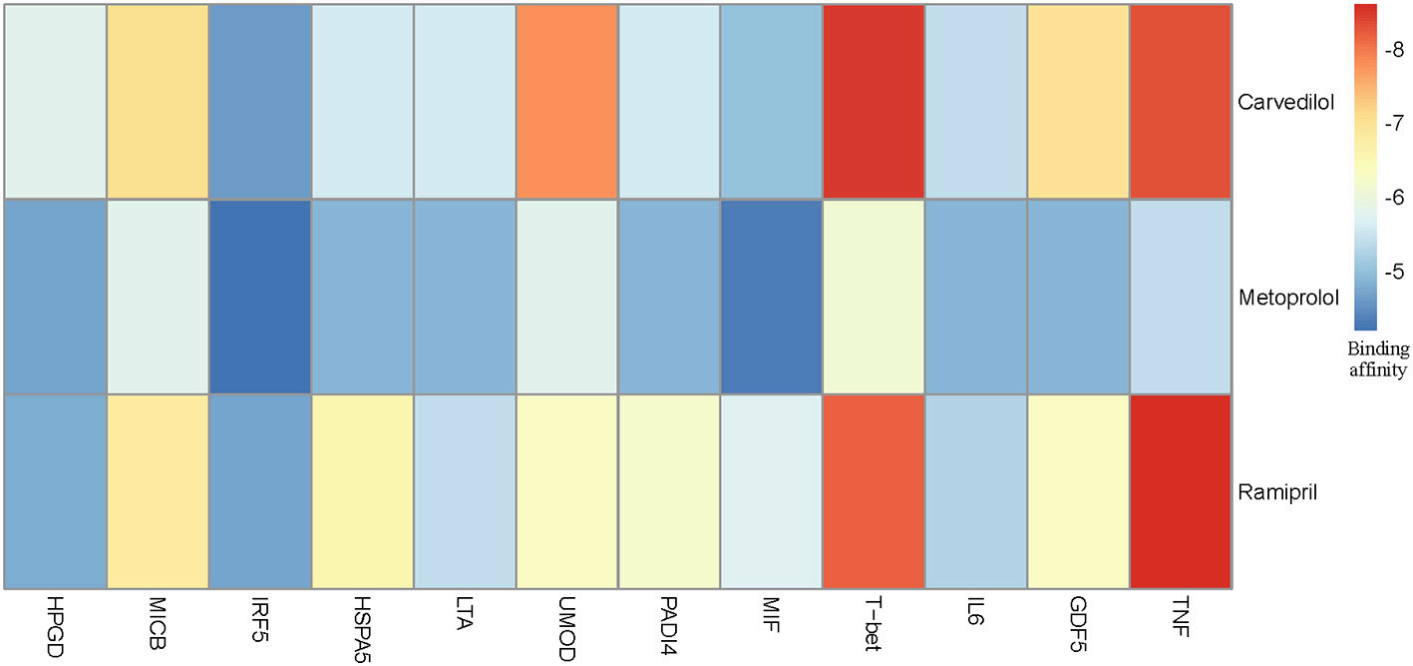
Molecular docking results between three drug molecules (Carvedilol, Metoprolol, and Ramipril) and Rheumatoid Arthritis.

We chose three drugs, Carvedilol, Metoprolol, and Ramipril, from Table 4. The *Drug_score* of these three drugs are all 0.8, which means they can treat four cardiovascular diseases in CMD 7 according to the records in CTD. The one remaining disease with no relevant records in CTD is Rheumatoid Arthritis (RA). We carried out molecular docking experiments using AutoDock Vina (Trott and Olson, 2010) to verify the three drugs. AutoDock Vina is a suite of docking tools, which is designed to predict how small molecules, such as substrates or drug candidates, bind to a receptor of known 3D structure. We downloaded drugs or molecules information from DrugBank database (Wishart et al., 2006) (https://www.drugbank.ca/) as ligands. The protein PDB files of diseases were obtained from RCSB PDB database (Deshpande et al., 2005) (http://www.rcsb.org/pdb/home/home.do) as receptors. We used these three drug molecules and RA related proteins for molecular docking. The results are shown in Figure 5. Binding affinity represents the strength of the binding interactions between the causal proteins of RA to the three drugs, Carvedilol, Metoprolol, and Ramipril (Gohlke and Klebe, 2002). Binding affinity is translated into physico-chemical terms in the equilibrium dissociation constant (KD) (Azimzadeh and Van Regenmortel, 1990), which is used to evaluate and rank order strengths of bimolecular interactions. The smaller the KD value, the greater the binding affinity of the ligand for its target. The results in Figure 5 showed that the three drug molecules, Carvedilol, Metoprolol, and Ramipril, can be well-docked with the casual proteins of RA.

#### Possible Treatment Mechanism for RA

**Figure 6:**
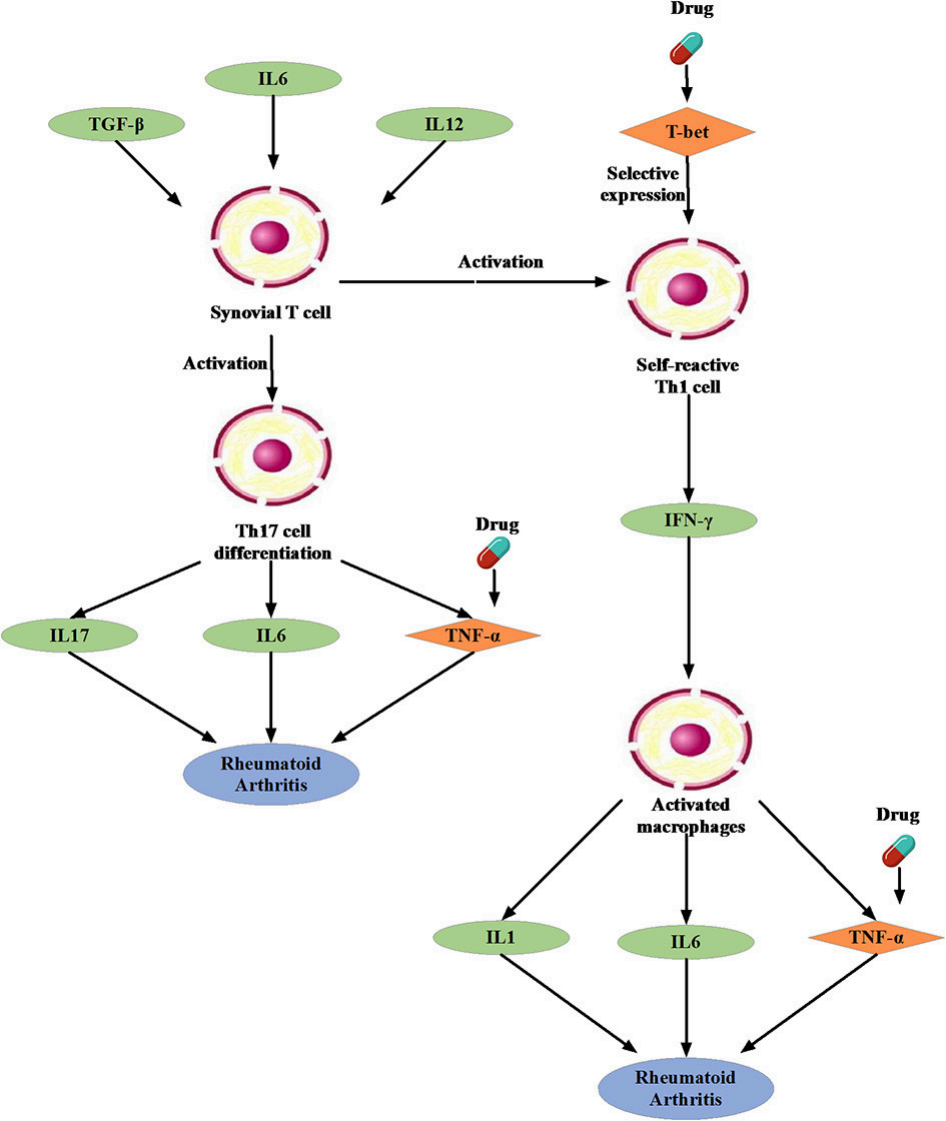
The possible treatment mechanism that drugs affect Rheumatoid Arthritis. The diamond is the potential targets gene. The green oval represents the intermediate gene that involved in the regulation process. The circle represents a specific cell. The blue oval represents the Rheumatoid Arthritis.

We noted that the binding affinity between each drug molecule with T-bet and TNF are all smaller (marked as red rectangle in Figure 5). We inferred that the three drugs more likely treated RA by affecting T-bet and TNF. Figure 6 gave the possible treatment mechanism that drugs affect Rheumatoid Arthritis. Synovial T cells may be activated by the combined action of TGF-β, interleukin 6 (IL6), and interleukin 12 (IL12) (McInnes and Schett, 2007). The activated synovial T cells possibly activate the differentiation of T-helper 17 (TH17) cells on the one hand and participate in the activation of T-helper 1 (TH1) cells on the other hand (McInnes and Schett, 2007). Both Th17 and Th1 cells belong to helper T cells, which are important regulatory and effector cells in the immune response. In fact, TNF has been reported that it is associated with the pathogenesis of Rheumatoid Arthritis (McInnes and Schett, 2011).

Moreover, many studies have been reported that Carvedilol and Ramipril have an effect on RA. Arab and El-Sawalhi (2013) pointed out that as a potential anti-arthritic drug, Carvedilol may be effect on the reduction of leukocyte migration. Fahmy Wahba et al. (2015) provided us a clue that Ramipril may represent a new promising strategy against RA because of its anti-inflammatory effect on rats. In short, it is very feasible to apply the conserved disease modules found by our method to drug repositioning research.

## Methods

### Constructing Disease Networks GODN and DODN

**Figure 7:**
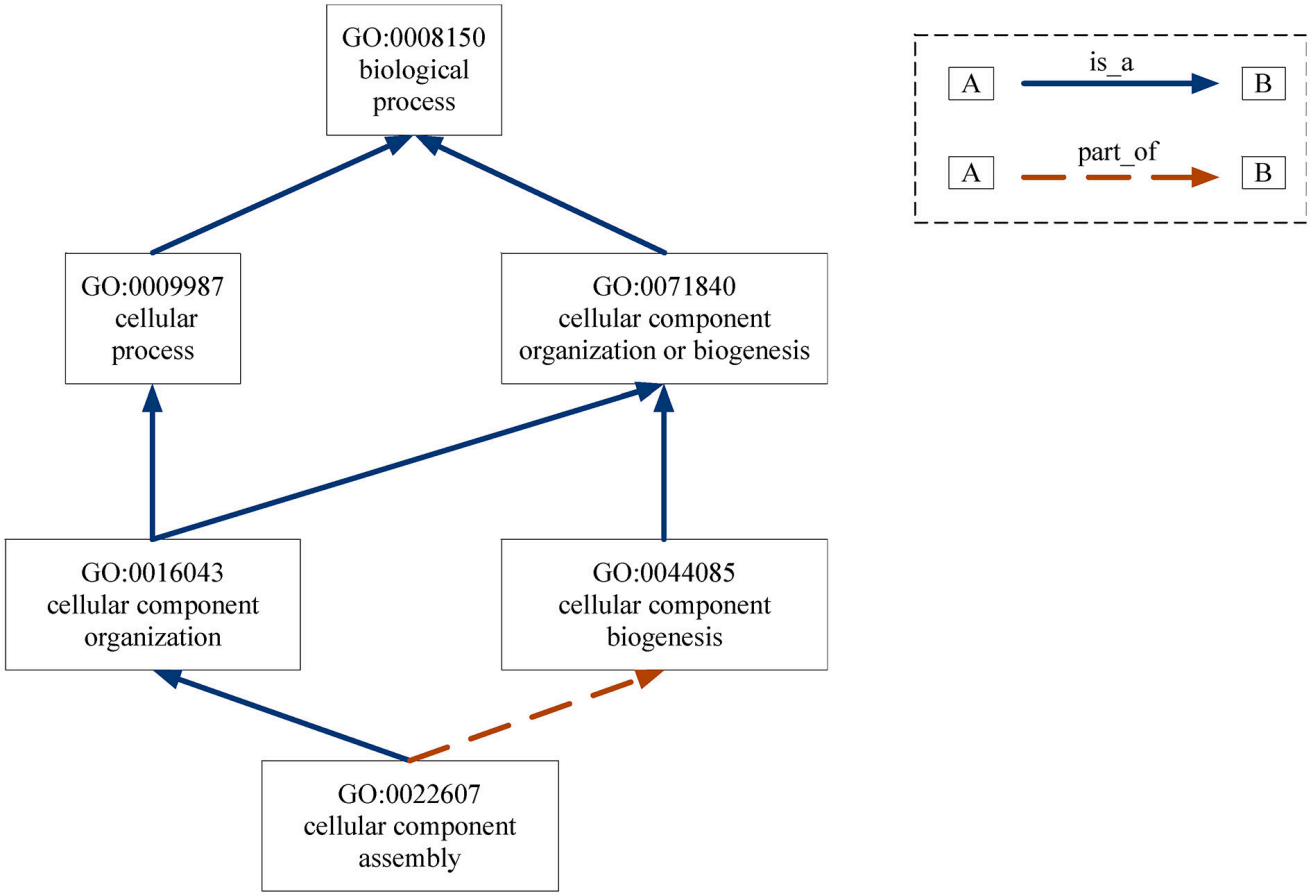
DAG for GO term “cellular component assembly:0022607”. Nodes represent the GO terms and edges represent the “is_a” and “part_of” relationships between terms.

Gene Ontology (GO) provides the consistent representations of gene products across databases (Ashburner et al., 2000). The categories in GO can be described as directed acyclic graphs (DAGs) (Thulasiraman and Swamy, 1992). Nodes represent the terms and edges represent the two kinds of semantic relations (“is_a” and “part_of”). The “is_a” relation forms the basic structure of GO. A “is_a” B means node A is a subtype of node B. The relation “part_of” is used to represent part-whole relationships in GO. Figure 7 gives an example of the DAG for GO term “cellular component assembly: 0022607.” There are six GO terms and seven relations between them in Figure 7. The solid blue arrow represents the “is-a” relation and the dotted brown arrow represents the “part-of” relation.

Based on DAGs, Wang et al. (2007) proposed a method to calculate the functional similarities of genes based on gene annotation information in GO database. For term *i* and term *j* in GO, the semantic similarity between them is defined as below (Wang et al., 2007):

(3) $$S_{GO}\left(i,j\right)=\frac{\sum_{t\in T_{i}\cap T_{j}}\left(S_{i}\left(t\right)+S_{j}\left(t\right)\right)}{SV\left(i\right)+SV\left(j\right)}$$

where $S_{{}^{*}}$(*t*) [defined by formula (4) (Wang et al., 2007)] indicates the contribution of term *t* to term “^*^”; $T_{{}^{*}}$ is a GO term set, including term “^*^” and all of its ancestor terms in the DAG; *SV*(^*^) [defined by formula (5) (Wang et al., 2007)] describes semantic similarity of GO term “^*^.” For any*t* ∈ *T*_*i*_, its contribution to term *i, S*_*i*_(*t*), can be defined as Wang et al. (2007):

(4) $$\{\begin{array}{l} S_{i}\left(t\right)=1\text{ if }t=i;\\ S_{i}\left(t\right)=\max\left\{w_{e}*S_{i}\left(t^{\prime }\right)|t^{\prime }\in childernof\left(t\right)\right\}\text{ if }t\neq i \end{array}$$

where *w*_*e*_ is the semantic contribution factor (0 < *w*_*e*_ < 1); *e* ∈ *E*_*i*_links term *t* with its child term *t*′; *E*_*i*_ is the edge set connecting the terms in the DAG for i. From formula (4), we can find that the contribution of term *i* to itself is 1. Other terms' contributions to term *i* are decreasing as the distance increases. The semantic similarity of GO term *i, SV*(*i*), can be got based on formula (5). Its definition is shown as follows (Wang et al., 2007):

(5) $$SV\left(i\right)=\sum_{t\in T_{i}}S_{i}\left(t\right)$$

According to the formulas (3–5), we can calculate the similarity between two GO terms *i* and *j*. Based on these, we can further calculate the similarity between two sets of terms *G*_1_ and *G*_2_ as Wang et al. (2007):

(6) $$Sim\left(G_{1},G_{2}\right)=\frac{1}{\left|G_{1}\right|+\left|G_{2}\right|}\times \left(\sum_{s\in G_{1}}sim\left(s,G_{2}\right)+\sum_{t\in G_{2}}sim\left(t,G_{1}\right)\right)$$

where |*G*_1_| and |*G*_2_| represent the numbers of terms in *G*_1_ and *G*_2_, respectively;*sim*(*s, G*_2_) represents the maximum of similarity between term *s* with any term in set *G*_2_, i.e., $sim\left(s,G_{2}\right)=\max_{t^{\prime }\in G_{2}}S_{GO}\left(s,t^{\prime }\right);sim\left(t,G_{1}\right)$represents the maximum of similarity between term *t* with any term in set *G*_1_, i.e.,$sim\left(t,G_{1}\right)=\max_{t^{\prime }\in G_{1}}S_{GO}\left(t,t^{\prime }\right)$.

**Figure 8:**
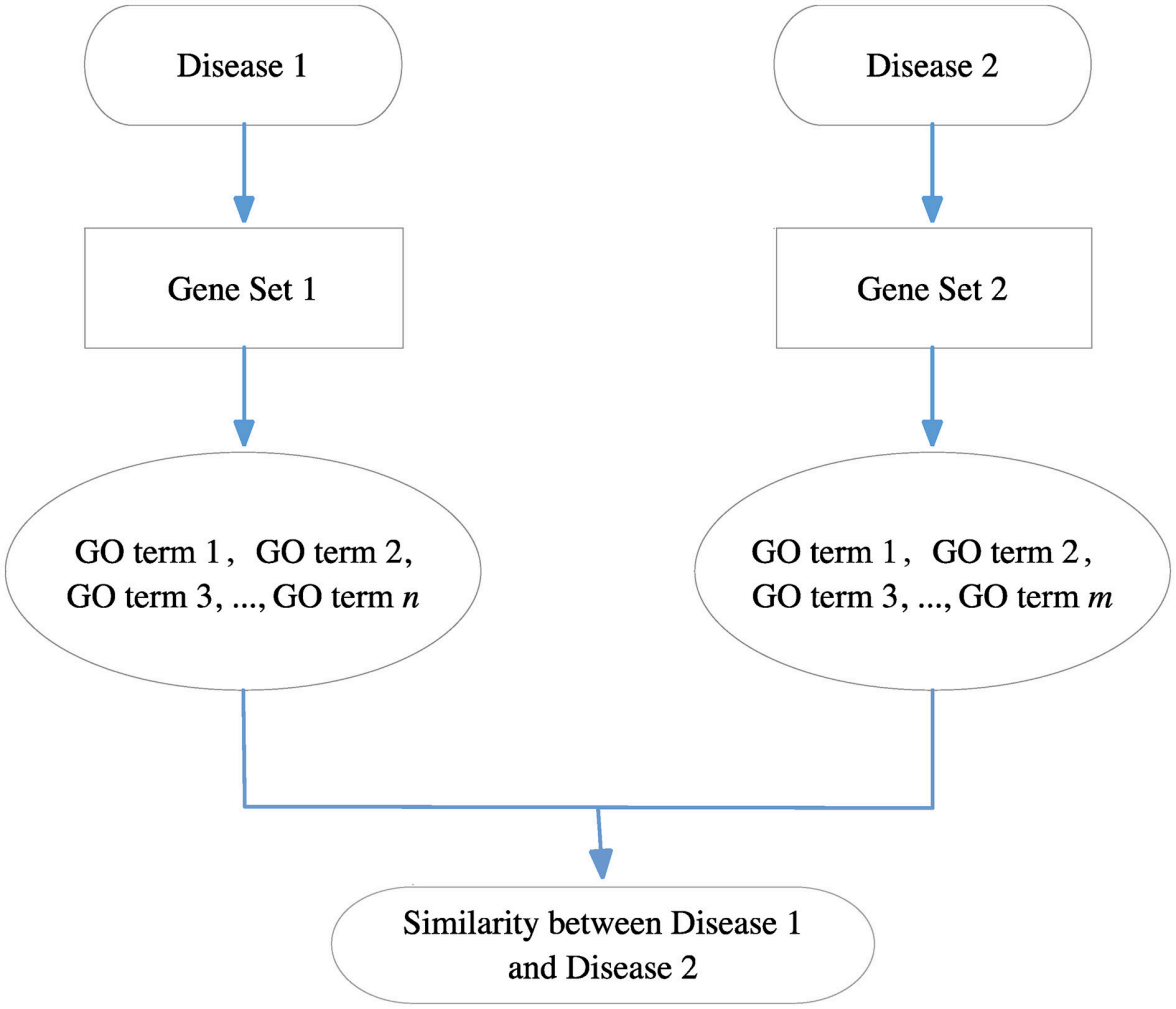
The computational framework of disease similarities based on GO terms. For each disease pair, we can get their related gene sets separately and then map them to the GO terms. Finally, we get two GO term sets. We can calculate the similarity between the two GO terms to obtain the relationship of the two diseases.

Because each disease relates to a gene set and each gene set can be mapped to a GO term set, we can evaluate the correlation between two diseases based on the similarity between their related GO term sets. Figure 8 gives the computational framework of disease similarities based on GO terms. In this way, we can construct the GODN.

**Figure 9:**
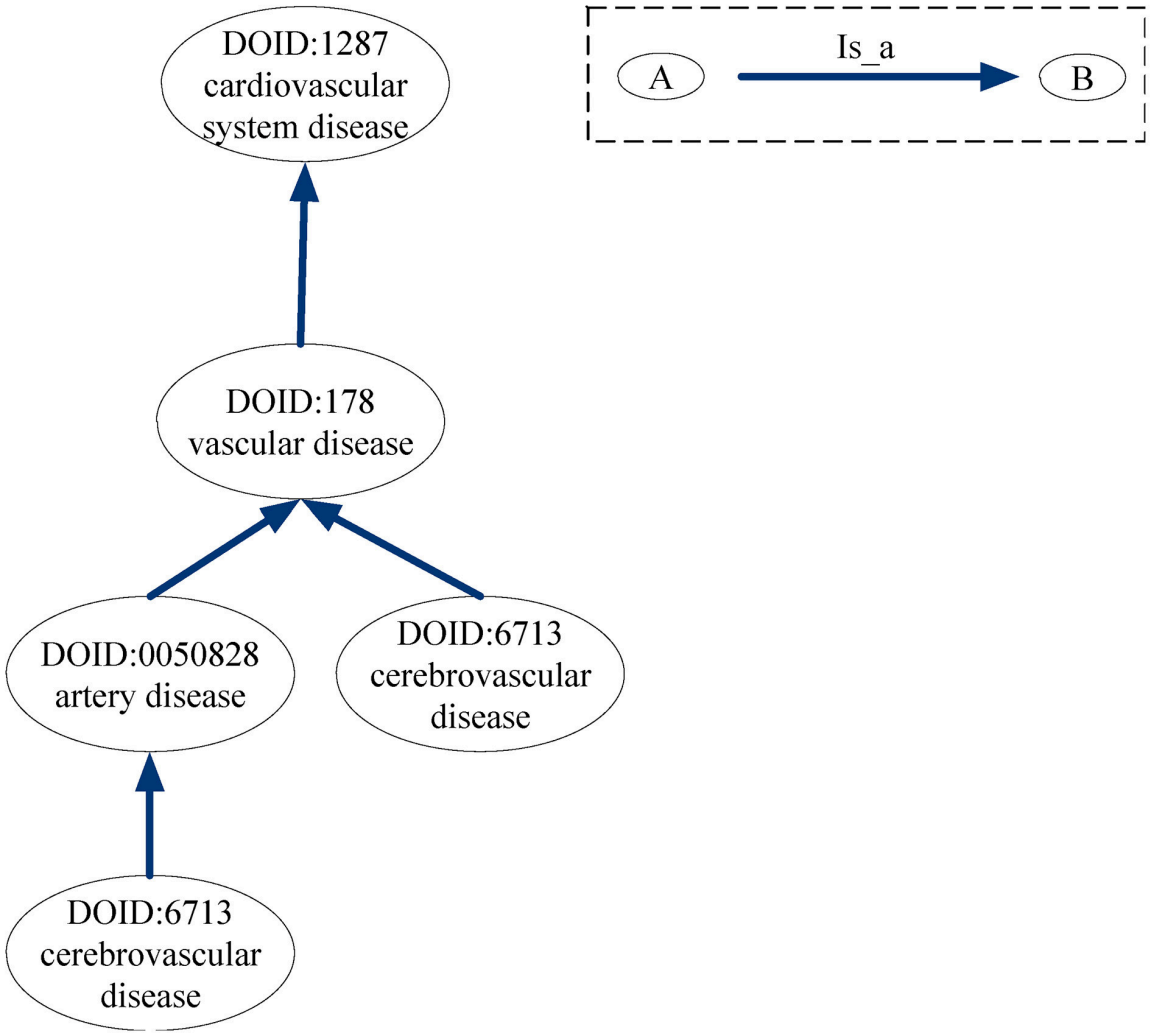
DAG for DO term “cerebrovascular disease: 6713.” Nodes represent the DO terms and edges represent the “is_a” relationships between terms.

For the DO-based disease similarity network (DODN), the constructing process is similar to that of GODN. Disease Ontology (DO) is a standardized ontology with consistent, reusable and sustainable descriptions of human disease terms (Schriml et al., 2011). Similar to GO, the associations between disease terms in DO can also be presented as DAGs (Thulasiraman and Swamy, 1992). Figure 9 gives an example of the DAG for DO term “cerebrovascular disease: 6713.” Nodes represent the DO terms and edges represent the “is_a” relationships between terms. For instance, DO term “cerebrovascular disease: 6713” is a subclass of DO term “artery disease: 0050828.” As a result, each disease corresponds to a DO term set. In Figure 9, DO term “cerebrovascular disease: 6713” corresponds to a set {“cerebrovascular disease: 6713,” “artery disease:0050828,” “cerebrovascular disease: 6713,” “vascular disease: 178,” “cardiovascular system disease: 1287”}. The similarity between two DO terms represents the relationship between two diseases. Therefore, we use the same method (Wang et al., 2007) as GODN to construct DODN.

### Extracting Conserved Modules From the Four-Layer Disease Network

The method (Li et al., 2011) for extracting conserved modules from the four-layer disease network is based on tensor analysis for multi-networks, which describes the multi-layer complex network as a third-order tensor:

(7) $$A={\left(a_{ijk}\right)}_{n\times n\times m}$$

where *a*_*ijk*_ represents the weight of the edge between disease *i* and disease *j* in layer *k*; *n*, and *m*, respectively, represent the number of diseases in each layer and the number of layers. The modules in single-layer networks are considered to be tightly internal connections and loosely external connection, which also can be extended to multi-layer networks, such as multi-layer disease networks. Here, we call the modules appear in the four-layer disease network as *conserved disease modules* (CDMs). The nodes of a CDM are the same in each occurrence, but the edge weights may vary between networks. The sum of edge weights in the CDM can be defined as Li et al. (2011):

(8) $$H_{A}\left(x,y\right)=\frac{1}{2}\sum_{i=1}^{n}\sum_{j=1}^{n}\sum_{k=1}^{m}a_{ijk}x_{i}x_{j}y_{k}$$

where *x* = (*x*_1_, …, *x*_*n*_)^*T*^ represents disease membership vector and *n* is the number of diseases in each layer. If disease *i* appears in the CDM, *x*_*i*_ = 1; otherwise, *x*_*i*_ = 0. *y* = (*y*_1_, …, *y*_*m*_)^*T*^ represents the network membership vector and *m* is the number of disease networks. Here, *m* = 4. If the CDM appears in network *j, y*_*j*_ = 1; otherwise, *y*_*j*_ = 0. Because CDMs represent the disease modules appearing in all the four networks, *y*_*j*_ = 1 in our work. Discovering conserved disease modules can be formulated by a discrete combinatorial optimization problem (Li et al., 2011): among all CDMs of fixed size, we look for the heaviest, i.e., the maximum of *H*_*A*_, which can be converted to a continuous optimization problem expressed as following (Li et al., 2011):

(9) $$\begin{array}{l} \max_{x\in {\mathbb{R}}_{+}^{n},y\in {\mathbb{R}}_{+}^{m}}H_{A}\left(x,y\right)\\ subject\;to\;\{\begin{array}{l} f\left(x\right)=1\\ g\left(y\right)=1 \end{array} \end{array}$$

where ℝ_+_ is a non-negative real space; *f* (*x*) and *g*(*y*) are vector norms. These equations give a tensor-based computational framework and we use it to identify CDMs. The size of CDMs is set to be no < 5 and the sum of edge weights in CDMs is set to be no < 0.3.

## Discussion

The framework of multi-layer network in this work is motivated by the underlying disease relationship at different levels. Considering the multidimensional information of the disease, we first constructed four disease similarity networks, namely, PIDN, DSDN, GODN, and DODN. Then, we integrated these four disease similarity networks to get a four-layer disease network. Based on the four-layer disease network, we obtained nine conserved disease modules by tensor-based computational framework. The sizes of these nine disease modules range from 5 to 17. We classified the disease modules based on the MeSH database and used 0.6 as threshold to determine the classification of a disease module. Diseases in conserved modules mostly belonged to a same category. For those diseases whose classification are different from others are more likely the potential disease-disease relationship.

We verified the reliability of our results from a statistical point of view. We randomly disturbed the edges of four disease networks to ensure that the degree of nodes remained unchanged. After repeating the above procedure for 1,000 times, we did not find any conserved disease module. We constructed a statistical experiment by using a disease similarity network as a standard dataset which was created by van Driel et al. (2006), named as Van's network. We firstly found the nine conserved disease module from Van's network and summed weights of each modules. Then we compared the sums with random modules extracted from Van's network. We repeated the above procedure for 10,000 times and found the *p*-values were lower than 0.001. We also made a comparison with the results of single-layer network clustering and found that modules exacted from multi-layer network were more reliable and accurate.

We used the pathogenic genes of each disease in conserved disease module 1 for KEGG enrichment analysis and found many pathways significantly enriched with most of diseases, such as hsa05320, hsa05332, hsa04612, hsa05202, hsa04380, and hsa04060. Through frequency analysis of pathogenic genes in disease similarity module 1, we found that TNF (tumor necrosis factor) gene had the highest frequency. As reported,^1^ TNF plays an important role in fighting against pathogens and tumor. It acts via the tumor necrosis factor receptor (TNFR) for triggering apoptosis. For diseases in module 1, TNF maybe their potential causal gene or have close connections with their casual genes, which will be useful for studying the mechanism of these diseases.

More importantly, our method can find potential disease-disease relationships. Taking conserved disease module 3 as a case, we found lupus erythematosus is an immune system disease that did not has the same classification as others, i.e., male urogenital disease. However, lupus erythematosus shared a lot of drugs with other diseases in module 3 for treatment which suggested that we found the potential relationship between lupus erythematosus and other diseases. As an application of our finding, we can reposition drugs among diseases in a same module. Taking conserved disease module 7 as a case, we found three potential drugs for Rheumatoid Arthritis (RA) based on molecular docking experiments. Furthermore, literature verification was also made.

In summary, our model for constructing multi-layer disease network can get more accurate conserved disease modules. As mentioned above, we verified our results from many aspects. However, there are still some shortcomings. Since our results are data-dependent, the incompleteness of the data affects the extracted module information. For example, DSDN network is relatively sparse comparing with other three networks due to preprocessing. In order to improve the quality of data, we need to filter false positive information in advance. This lead to data scale reduction. Based on such data, we may only find some of the meaningful results. As the data continues to improve, we will find more and more meaningful conserved disease modules. In addition, in the framework of a multi-layer network, more categories of disease data can be integrated, which will help to do more in-depth research on disease mechanisms.

## Author Contributions

LY and YZ contributed conception and design of the study. SY organized the database and performed the experiments. LY and YZ performed the results analysis. LY wrote the first draft of the manuscript. LG, YZ, and SY wrote sections of the manuscript. All authors contributed to manuscript revision, read, and approved the submitted version.

### Conflict of Interest Statement

The authors declare that the research was conducted in the absence of any commercial or financial relationships that could be construed as a potential conflict of interest.

## Footnotes

^1^University of Erlangen-Nuremberg. “How tumor necrosis factor protects against infection.” ScienceDaily. ScienceDaily, 11 July 2016.

## Supplementary Material

Table S1 Disease-gene data combining GWAS and OMIM databases.

Table S2 Human disease similarity data based on disease symptoms.

Table S3 Human diseases similarity data based on GO terms.

Table S4 Human diseases similarity data based on DO terms.
